# Analytical modelling of temperature effects on an AMPA-type synapse

**DOI:** 10.1007/s10827-018-0684-x

**Published:** 2018-05-11

**Authors:** Dominik S. Kufel, Grzegorz M. Wojcik

**Affiliations:** 1 The Polish Children’s Fund, Pasteura 5a, 02-093 Warsaw, Poland; 2 Department of Physics & Astronomy, University College London, 0 Gower St, WC1E 6BT London, UK; 3 Faculty of Mathematics, Physics and Computer Science, Institute of Computer Science, Department of Neuroinformatics, Maria Curie-Sklodowska University in Lublin, Akademicka 9, 20-033 Lublin, Poland

**Keywords:** Temperature, AMPA-receptor, Modeling of synapses, Analytical modeling, Uncoupling set of ODEs

## Abstract

It was previously reported, that temperature may significantly influence neural dynamics on the different levels of brain function. Thus, in computational neuroscience, it would be useful to make models scalable for a wide range of various brain temperatures. However, lack of experimental data and an absence of temperature-dependent analytical models of synaptic conductance does not allow to include temperature effects at the multi-neuron modeling level. In this paper, we propose a first step to deal with this problem: A new analytical model of AMPA-type synaptic conductance, which is able to incorporate temperature effects in low-frequency stimulations. It was constructed based on Markov model description of AMPA receptor kinetics using the set of coupled ODEs. The closed-form solution for the set of differential equations was found using uncoupling assumption (introduced in the paper) with few simplifications motivated both from experimental data and from Monte Carlo simulation of synaptic transmission. The model may be used for computationally efficient and biologically accurate implementation of temperature effects on AMPA receptor conductance in large-scale neural network simulations. As a result, it may open a wide range of new possibilities for researching the influence of temperature on certain aspects of brain functioning.

## Introduction

From a medical perspective, it has been suggested that tight control of brain temperature in patients, suffering during a post-traumatic period is highly recommended (Shigemori et al. 2012). However, despite the fact that the techniques of the control of the brain temperature have been developed, direct mechanisms of the influence of temperature on neural dynamics are still uncertain (Badjatia 2009). Better understanding of temperature effects on different levels of brain function may be useful in developing more sophisticated methods of treatment for different neurological disorders which are sensitive to temperature (including hot-water epilepsy (Kowacs et al. 2005), autism (Helt et al. 2008) or brain injury (Mrozek et al. 2012; Dietrich 1992).

On the level of single neurons, multiple effects of temperature on brain function have been observed. The most important from the perspective of neural dynamics are:

1. Temperature influences membrane resting potential (Hodgkin and Huxley 1952; Buzatu 2009).
2. Temperature affects ion channels dynamics (Hille 2001; Sterratt 2015).
3. Temperature affects synaptic transmission (Asztely et al. 1997; Weight and Erulkar 1976; Schiff and Somjen 1985).

Temperature effects on membrane resting potentials (the Goldman-Hodgkin-Katz equation) and on ion-channel dynamics (e.g. Hodgkin and Huxley (1952)) are now relatively well-characterized. But the influence of temperature on synaptic transmission has proven to be more difficult to model (e.g. De Schutter et al. (2009)). This may be because of the various processes involved in synaptic transmission, such as presynaptic release of neurotransmitter; dynamics of a vesicle pore; diffusion of neurotransmitter; binding of the neurotransmitter; and kinetics of postsynaptic receptors. The influence of temperature on each of this processes is different, and they combine to the significant modification of the synaptic transmission (Fuxe et al. 2005).

Therefore, generally, in the creation of biologically-real models in computational neuroscience, it is useful to make them easily scalable for different temperatures. This is especially important because some of the neurobiological experiments - for example, *in vitro* studies - are usually conducted at temperatures lower than physiological ones. Therefore, a better knowledge about the temperature dependence of synaptic transmission may be crucial for linking *in vitro* and *in vivo* studies. Furthermore, an optimal way of including a full description of temperature effects in neural simulations may allow computational brain research to address new areas: for example, investigation of the influence of temperature on such neurological disorders like hot-water epilepsy, cerebral brain injury or autism.

It is possible to tackle the problem of including temperature effects on synapses from different perspectives:

(1): A first approach is to include some multiplicative coefficients accounting for the influence of temperature in phenomenological synapse models e.g. alpha function, dual-exponential functions, single exponential functions. In this approach, it is required to multiply all of these time constants and amplitudes of phenomenological functions by some (probably different) factors associated with temperature. These coefficients would mimic the increase in the speed of chemical reactions with temperature (according to Arrhenius equation). However, there is a significant problem related to this approach. The values of temperature multiplicative factors are not known *a priori* (for an arbitrarily chosen kinetic scheme). These values would have to be obtained for each study separately by performing additional neurobiological experiments at different temperatures, which is usually not possible for *in vitro* research.
(2): A second approach is to model synapses on the microphysiological level - to investigate temperature effects on the kinetics of synaptic receptor proteins, with conformation dynamics described by kinetic schemes. To include temperature effects, it is required to multiply all of the kinetic rate constants between different conformational states by coefficients dependent on temperature. This approach was previously taken experimentally (Postlethwaite et al. 2007; Cais et al. 2008). Nonetheless, the possible problem is that all of the temperature coefficients (which scale rate constants) are specific for given kinetic scheme. So, even if temperature coefficients in one kinetic scheme were found, they would be invalid for other schemes (unless one finds a way to link different kinetic schemes, which is currently not possible apart from linking very simple kinetic models (Shelley and Magleby 2008)).

In fact, both of the approaches described above are similar, as amplitude and time constants in phenomenological modeling (under certain assumptions) may be interpreted as a combination of different kinetic rates (Destexhe et al. 1994b).

Generally, the problem of including temperature effects in synapse modeling is complex and yet not well-characterized. Both the first and second approaches are hard to generalize for different phenomenological functions describing synaptic conductance or for different microphysiological kinetic schemes. Including temperature effects, require additional neurobiological experiments, which does not allow previously developed models to be easily scalable for a wide range of brain temperatures. In this paper, a novel approach to the problem of including temperature effects on modeling synapses is proposed. On the basis of previous experimental and numerical research, we construct assumptions for a new analytical model to include temperature effects in modeling the kinetics of the *α*-amino-3-hydroxy-5-methyl-4-isoxazolepropionic acid (AMPA) receptor. First, using Monte Carlo simulation and Markov modeling we propose simplifications of an experimental kinetic scheme (Postlethwaite et al. 2007) to allow for a closed form solution of the set of ODEs describing this problem. Second, we introduce the concept of uncoupling of the differential equation system describing AMPA receptor kinetics. Third, after solving the resulting set of differential equations, we compare results using the constructed model with numerical and experimental data. Finally, we suggest that our model provides a simple way to mimic temperature effects in neural dynamics simulations at low frequencies, regardless of the phenomenological function used to describe AMPA synaptic conductance.

## Methods and model

In this section, we construct a new, analytical model of the conductance of AMPA-type synapse for low-frequency stimulations using uncoupling assumption of set of ODEs along with simplifications from experimental and numerical data.

### **Monte Carlo simulation of synaptic transmission**

Some of the assumptions of the analytical model presented below are based on the analysis of the data from Monte Carlo simulation of the synaptic transmission. The simulation was constructed based on the assumptions and parameters of Postlethwaite et al. (2007) - code of their original simulation is available^1^ using the MCell simulator (Stiles et al. 1996).

The most important assumptions of the simulation are as follows:

1. The geometry used in the Monte Carlo simulation refers to the AMPA-type synapse at the calyx of Held of a rat. The morphological data was given by Sätzler et al. (2002). In the simulation, presynaptic and postsynaptic terminals were separated by the synaptic cleft of 28nm. Postsynaptic terminal consisted Postsynaptic Density (PSD) with an area of 0.32 micrometer on 0.32 micrometer, populated by 80 AMPA receptors. Additionally, four neighboring PSDs were included - separated by 317 nm.
2. Vesicle from which glutamate was released was a cube with the volume equal to the volume given by Sätzler et al. (2002) and connected to the synaptic cleft by a gradually opening (with exponential dynamics) fusion pore. Vesicle was released at variable locations above a central postsynaptic density (PSD). Each vesicle contained 6000 glutamate molecules.
3. The diffusion rate of the glutamate was assumed to be equal to 3 ⋅ 10^6^*cm*^2^/*s* for the receptor kinetic parameters detailed in Table 1.

**Table 1:** Parameters of the AMPA receptor model (see kinetic scheme and text)

**Parameter**	**Description**	**Value**
*k*_*b*_	Agonist binding rate	10^7^ [1/*molar* ⋅ 1/*s*]
*k*_*u*_	Agonist unbinding rate	8 ⋅ 10^3^ [1/*s*]
*k*_*o*_	Channel opening rate	20 ⋅ 10^3^ [1/*s*]
*k*_*c*_	Channel closing rate	10 ⋅ 10^3^ [1/*s*]
*k*_*d*_	Desensitization rate	4 ⋅ 10^3^ [1/*s*]
*k*_*r*_	Resensitization rate	15 [1/*s*]
*A*	Amplitude of glutamate concentration	7.48 ⋅ 10^−4^ [*molar*]
*ω*	Decay time constant of glutamate concentration	2471 [1/*s*]
*Q*_10_	Coefficient of temperature dependence of kinetic rates	2.4

All of the simulations used 1 ⋅ 10^−6^ *s* time step. For more detailed discussion about the assumptions of the Monte Carlo simulation see Postlethwaite et al. (2007). All standard parameters used in the simulation and further analytical model are presented in the Table 1. The code in MCell is available.^2^

### **Analytical model**

**Fig. 1:**
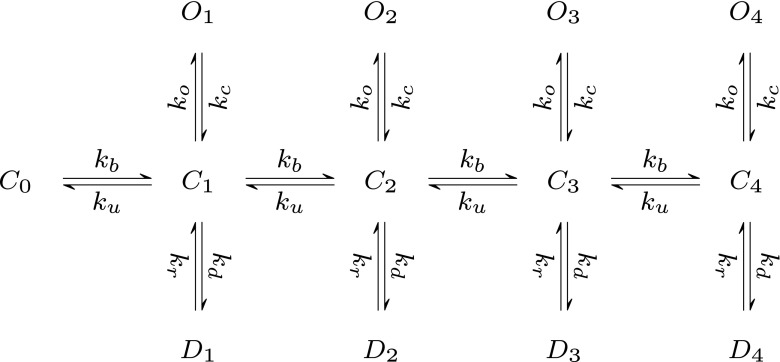
Scheme 1 - modified kinetic scheme model by Postlethwaite et al. (2007), independent binding was assumed (see below) and no transitions between desensitized states (with minor influence on accuracy of results)

**Fig. 2:**
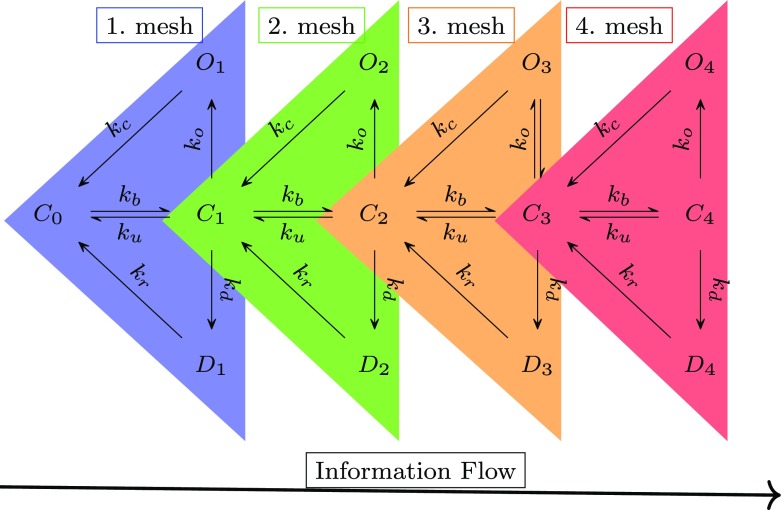
Scheme 2. Kinetic scheme used for construction of an analytical model consists five orders of subconductance (index numbers of states: 0,1,2,3,4) and four meshes (colored triangles)

Our model is based on the following numerical and experimental findings:

- **(1)** Acceleration in postsynaptic AMPA receptor kinetics is the predominant effect of increased temperature on altered synaptic responses at low frequencies ((Postlethwaite et al. 2007)). With this assumption, modeling of temperature effects on synapses was simplified by considering temperature effects only on AMPA receptor kinetics, rather than also on modified presynaptic release and/or neurotransmitter diffusion dynamics. Furthermore, we assumed that to include temperature effects on AMPA receptor kinetics it is sufficient to multiply all of the rate constants (*k*_*b*_,*k*_*o*_,*k*_*c*_,*k*_*d*_,*k*_*r*_) by a single temperature coefficient *Q*_10_ (Postlethwaite et al. 2007).^3^
- **(2)** As suggested by Postlethwaite et al. (2007), temperature effects are mediated by driving AMPARs to higher sub-conductance states. To include higher sub-conductance states in an analytical model of AMPA receptors, a few simplifications of the complex 13 state, 30 transitions kinetic scheme of Postlethwaite et al. (2007) (Scheme 1 in Fig. 1) were made. Scheme 1 was re-written into the simplified form of Scheme 2. This form uses the symmetry of states and transitions in Scheme 1. Based on uncoupling of equations, described in point (4) below, this symmetry is evident in that one can divide Scheme 1 into five orders of sub-conducting states.
- **(3)** For simplification all of the state transitions except the transition from closed to bound states in AMPA receptor kinetics (considering the single mesh of Scheme 2 in Fig. 2) were assumed to be Markov models: time and voltage independent and dependent only on the occupancy of neighboring states as was previously proposed by Destexhe et al. (1994b).

Additional simplification was as follows: modifications in the directions of the transitions with rates *k*_*r*_ and *k*_*c*_ were made to rewrite Scheme 1 to Scheme 2 (these transitions are diagonal in Scheme 2 vs vertical in Scheme 1). This change simplified the dynamics of each closed (*C*) state by reducing its coupling with the adjacent open and desensitized states of the AMPA receptor. This approach allows for a much easier analytical solution of the differential equations describing the simplified kinetic scheme.

**Fig. 3:**
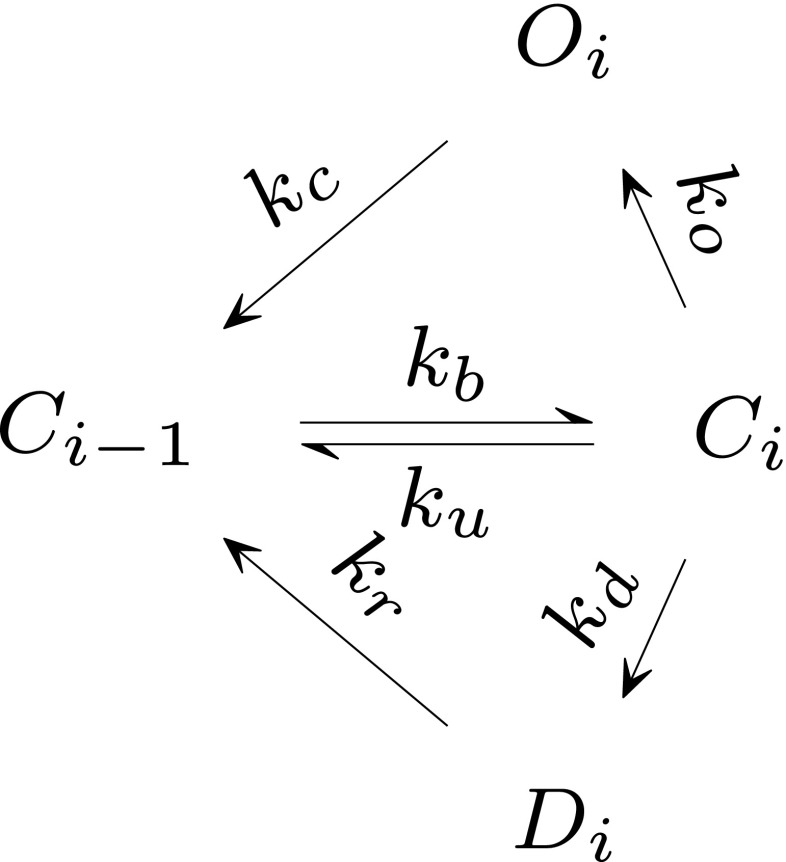
Scheme 3. Single mesh triangle from Scheme 2 described by an independent pair of coupled differential equations

**Fig. 4:**
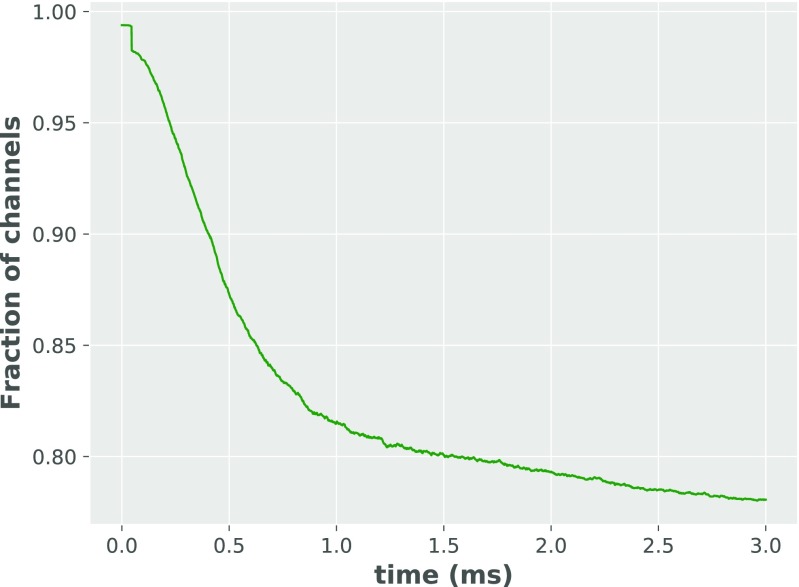
Fraction of AMPAR channels in unbound state *C*_0_. Fraction of channels decays from 1 to about 0.8 in 3*ms*, supporting our assumption that the fraction of channels in state *C*_*i*− 1_ in first mesh is far larger than the fraction of channels in the other states

Separately, the 1st order (and symmetrically, with additional assumptions described below, the i-th order) of the AMPAR kinetic states was assumed to behave as a single mesh, according to Scheme 3 in Fig. 3. It was assumed that *C*0 ≫ 1. This is the case when very few receptors bind glutamate so that nearly all receptors remain in form *C*_0_. This is motivated by comparing the number of channels in different states in the more detailed Monte Carlo simulation described above (see Fig. 4). Therefore, the fraction of channels in *C*_0_ state is considered always to be 1. In a further analogous simplification, each state in the i-th mesh, with *i* = 1,2,3,4, is likewise assumed to remain at 1. This assumption is necessary for an analytical model because otherwise, the system of differential equations is too complex to be solved analytically. Only the open states of the AMPA receptor (*O*_1_,*O*_2_,*O*_3_,*O*_4_) contribute to the synaptic conductance (Smith et al. 2000).

- **(4)** To describe the kinetic scheme with m states using differential equations it is necessary to write *m* − 1 coupled differential equations, which complexity is proportional to the number of transitions between different states (Destexhe et al. 1994b). With the purpose to simplify this description we introduce the assumption of uncoupling. In mathematical terms, assumption of uncoupling the differential equations for each single mesh may be written, for i-th order, as:
  1 $$ k_{b}(t) (x_{i-1}+x_{i}) \gg k_{c} y_{i + 1} + k_{r} z_{i + 1} + k_{u} (x_{i + 1}+x_{i}) $$
  Particulary, for 1st order we obtain:
  2 $$ k_{b}(t) (x_{0} + x_{1}) \gg k_{c} y_{2} + k_{r} z_{2} + k_{u} (x_{2}+x_{1}) $$
  where *x*_*i*_, *z*_*i*_, *y*_*i*_ are the fractions of channels in a state *C*_*i*_, *D*_*i*_, *O*_*i*_ respectively.
  We see that from the perspective of the (i + 1)-th order sub-conductance state, the fraction of channels in the i-th order sub-conductance bound state (*C*_*i*_) is perceived as 1 [generalization of assumption (3)]. However, we do introduce a scaling function to differentiate the absolute values of fractions in these *C*_*i*_ states. For each i-th order of conductance *λ*_*i*_(*t*) function is introduced. *λ*_*i*_(*t*) scales relative fraction of channels in each state to an absolute (scaled identically to all of the orders of the kinetic scheme) fraction.
  This method allows us to uncouple set of twelve coupled differential equations with a complex formulation to set of 4 pairs of differential equations (coupled only in pairs, rather than between different orders of sub-conductance). Using this approach, we are able to include higher order sub-conductance states and, as a result, find an analytical solution of the problem.
- **(5)** Glutamate binding was assumed to be independent, similarly to model by Robert and Howe (2003) This departure from Postlethwaite et al. (2007) was motivated by a disproportionate increase of the complexity (in comparison to the gain in accuracy) of an analytical solution of a set of differential equations when assuming cooperative binding.
- **(6)** Glutamate concentration was assumed to be time-dependent according to a single exponential decay function (Scimemi and Beato 2009), with parameters fitted to a simulation of glutamate concentration in the synaptic cleft (and consequently at the PSD) in the Monte Carlo model of synaptic transmission. Therefore, the function describing the binding rate, from closed to bound states, has a form dependent on the concentration of glutamate at the PSD:
  3 $$ k_{b}(t)=k_{b} A e^{-\omega t} $$
  where the parameters *ω* [1/s] and *A* [molar] were fitted from averaging glutamate concentration (in the cleft) in Monte Carlo simulation of synaptic transmission.

Therefore, the function describing the binding rate, from closed to bound states, has a form dependent on concentration of glutamate at the PSD: with a time constant *ω* and amplitude *A* fitted to the numerical data from the Monte Carlo simulation.^4^

A dual-exponential function [in the form of *y* = (*Ae*^*αt*^ − *Be*^*βt*^)] was also used as a fitting method for glutamate concentration, but the resulting, more complex, system of equations could not be solved analytically, and this increase in complexity was not compensated for by a substantial gain in accuracy of the model in comparison to the single-exponential function.

**(7)**: According to experimental data, AMPA receptors are tetramers (Rosenmund et al. 1998). Conductance of AMPA receptor can be described as a sum of conductances of all orders of subconductance multiplied by different constants for different orders of states in kinetic scheme as suggested by Sahara and Takahashi (2001):
  4 $$ g(t) = g_{4} \sum\limits_{i = 1}^{n = 4} a_{i} y_{i}(t) $$
  where, *g*_4_ is a peak conductance of a channel in 4-fold bound state, *n* is a number of orders in kinetic scheme and *a*_*i*_, *y*_*i*_ are scalling factor and fraction of open channels in the i-th state respectively.

Conductances of different orders of states in kinetic scheme were set as fractions of the peak conductance at the 4-fold bound state *O*_4_ (*O*_1_: *a*_1_ = 0.1, *O*_2_: *a*_2_ = 0.4, *O*_3_: *a*_3_ = 0.7, *O*_4_: *a*_4_ = 1.0) as was proposed by Postlethwaite et al. (2007), and motivated by previous experimental work by Smith et al. (2000). This assumption suggests that we may break down the problem of finding the synaptic conductance into finding the sum of four functions (one for each order of sub-conductance, see Fig. 2).

Combining the above assumptions simplifies the original complex kinetic scheme, containing 13 states and multiple transitions (described by 12 coupled differential equations). Modification of directions of transitions (from *O*_*i*_ and *D*_*i*_) using the uncoupling concept splits the corresponding system of differential equations to four, one-side dependent and specular between themselves (see Fig. 2), single meshes as in Fig. 3. Furthermore, by assuming the fraction of channels in state *C*_*i*− 1_ for i-th order of scheme to be equal to 1 we are able to solve a coupled pair of differential equations within every single mesh. Thus, simplification leads to 4 independent pairs of coupled differential equations. For the first order of conductance we know the solution explicitly and for higher orders we use *λ*_*i*_(*t*) = *x*_*i*− 1_(*t*) (which is the solution in respect to the fraction of channels in state *C*_*i*− 1_ of pair of differential equation for (i-1)-th order), which assures information flow from lower to higher orders of AMPAR sub-conductance.

Making these assumptions, one obtains the general system of coupled linear ODEs, describing the 1st through 4-th orders of kinetic states (Scheme 2):

5 $$ \frac{dx_{i}}{dt}=k_{b} A e^{-\omega t} \lambda_{i} (t) - (k_{o}+k_{u}+k_{d})x_{i}(t) $$

6 $$ \frac{dy_{i}}{dt}=k_{o} x_{i}(t) - k_{c} y_{i}(t) $$

where *y*_*i*_(*t*) = [*O*_*i*_(*t*)] is a fraction of channels in an open state of i-th order, *x*_*i*_(*t*) = [*C*_*i*_(*t*)] is a fraction of channels in a bound state of i-th order, *λ*_*i*_(*t*) is a function to convert fraction of all channels to same absolute scale (not only relative for each order) - for the i-th order of Scheme 2 it equals to solution with respect to fraction of channels in state *C*_0_ of two differential equations of (i-1)-th order: *λ*_*i*_(*t*) = *x*_*i*− 1_(*t*). Using this approach we may include higher sub-conductance states of AMPA receptor with an analytical approach, due to the uncoupling of differential equations describing the kinetic scheme.

The above set of linear coupled ODEs [(5) and (6)] has a closed-form solution. For the first order, with boundary conditions *y*_1_(0) = 0 and *x*_1_(0) = 0 the solution is:

7 $$ y_{1}(t)=\frac{A k_{b} k_{o}}{S P} e^{-\omega t} + \frac{A k_{b} k_{o}}{R P} e^{-(P + \omega) t} - \frac{A k_{b} k_{o}}{R S} e^{-k_{c} t} $$

where *S* = *k*_*c*_ − *ω*, *P* = *k*_*d*_ + *k*_*o*_ + *k*_*u*_ − *ω*, *R* = −*k*_*c*_ + *k*_*d*_ + *k*_*o*_ + *k*_*u*_.

As it is possible to be seen, the first-order approximating function is a sum of exponents (as suggested by Destexhe et al. (1994b)).

The full solution for all orders of a kinetic scheme (Scheme 2) can be found in Appendix A.

## Results

### **Analytical model of AMPA receptor**

**Fig. 5:**
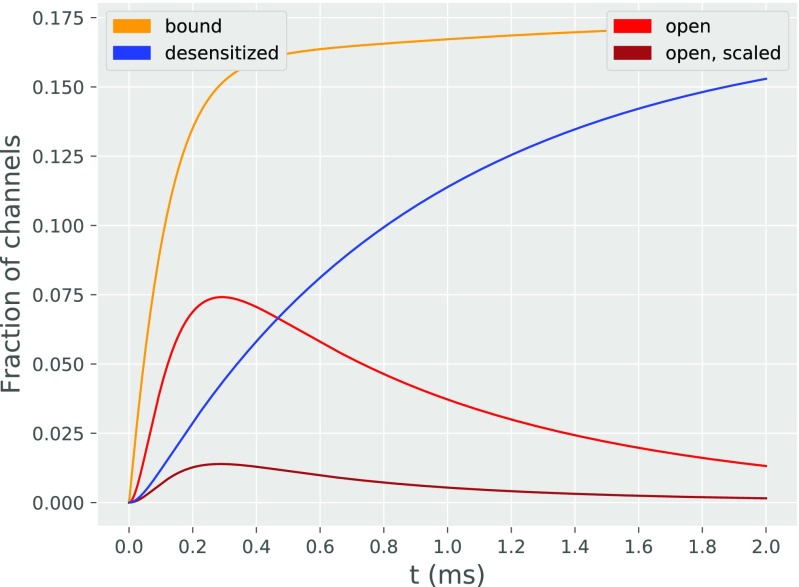
Fraction of channels in different states as a function of time. “Open, scaled channels” refers the sum of the fractions of channels in open states, with each fraction multiplied by its respective peak conductance

**Fig. 6:**
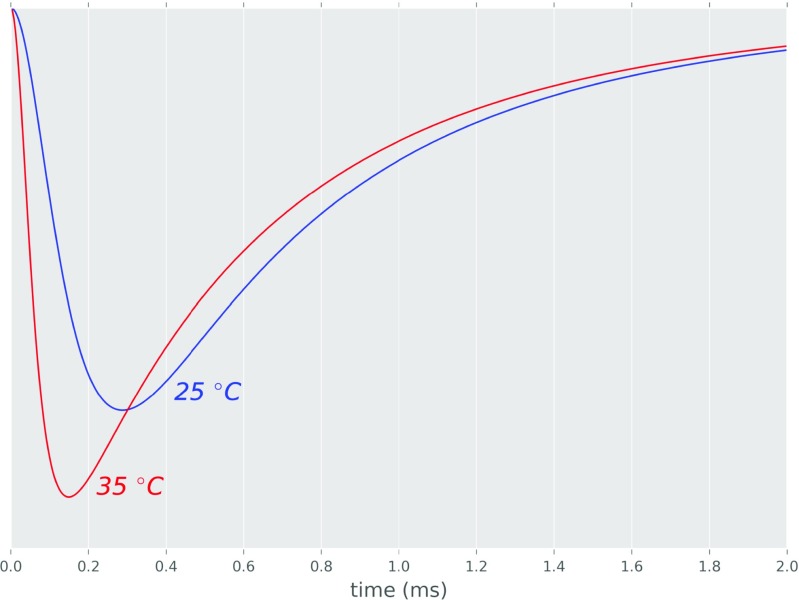
Conductance curve of AMPA synapse in 25 ^∘^C and 35 ^∘^C. Conductance in 35 ^∘^C in comparison to 25 ^∘^C has larger and quicker peak. Transition between 25 ^∘^C and 35 ^∘^C was achieved only by multiplication of all rate constants in the kinetic scheme by *Q*_10_ = 2.4

We found that, for kinetic rates fitted from the model of Postlethwaite et al. (2007), our analytical model is able to reproduce two of their key results from detailed Monte Carlo simulation. These results describe the dynamics of fractions of AMPAR channels in different states (compare Fig. 5 here with Fig. 2B of Postlethwaite et al. (2007)) and the time courses of AMPAR synaptic conductance at two different temperatures (compare Fig. 6 here with Fig. 1A of Postlethwaite et al. (2007)).

### **Fraction of channels**

Fractions of AMPAR channels in different states (Fig. 5) are both qualitatively and quantitatively (after normalization: see Discussion) consistent with this obtained in Monte Carlo simulation (with a mean error 5*%* for the time courses of the open states).

Just after the *t* = 0, binding of glutamate leads AMPARs to transition from unbound to bound states. The fraction of channels in bound states is dependent on glutamate concentration at PSDs and rate of unbinding in the AMPA receptor kinetic scheme. Excluding effects of unbinding, with the diffusion of neurotransmitters in synaptic cleft considered as a random walk, the mean distance of a single neurotransmitter from the location of release (vesicle pore) should increase over time proportionally to $\sqrt {N}$, where *N* is the number of steps). Hence, the glutamate concentration should decay with $1/\sqrt {N}$, and the number of bound states should increase proportionally to $1-\sqrt {N}$. However, as time passes some of the AMPAR channels unbind neurotransmitters (transitioning from *C*_1_ to *C*_0_ state), therefore eventually reaching equilibrium - so the function of bound state fraction (in time) should be close to a ‘flattened’ $1-\sqrt {N}$.

During synaptic transmission, AMPAR protein undergoes conformational changes. The rate of these changes is proportional to temperature - the effect of temperature is reflected in our analytical model by scaling all of the rate constants of the kinetic scheme by a *Q*_10_ parameter. The continuous growth of fraction of channels in desensitized states can be attributed to that the resensitizing rate of reaction is about three orders of magnitude smaller than the rate of desensitization. Therefore, channels after entering, are unlikely to leave desensitized states - the fraction of channels in desensitized states slowly approaches the fraction of channels in all bound states. Generally, the analytical model slightly underestimates (about 9% of the difference between analytical model and numerical results) the fraction of AMPAR channels in states that are bound and desensitized. This may come from the assumption in the analytical model about directions of transitions away from open states, which go from *O*_*i*_ states to *C*_*i*− 1_ states (rather than *C*_*i*_) and from *D*_*i*_ to *C*_*i*− 1_ states (rather than *C*_*i*_). Thus, in the first order of sub-conductance states (see Fig. 2), some transitions from the open state go back to the unbound state (rather than the first bound closed state as assumed in Scheme 1). Underestimation of the fraction of channels in desensitized states is due to the modification in directions of transitions for the first order of sub-conductance, smaller fraction of AMPARs is in a bound state. Resensitization (due to its low transition likelihood) has a minor influence on the results.

### **Synaptic conductance**

The AMPAR conductance curve (Fig. 6) obtained from the analytical model is able to reproduce (with 5% accuracy for the relative amplitude and peak time, which is within the experimental uncertainty range) the shape and scale of temperature effects on synaptic transmission (compared with Fig. 1B of Postlethwaite et al. (2007)). At 35 ^∘^C both the rise and decay time constants of synaptic conductance are smaller in value (peak time is shorter). The peak conductance is larger (ratio about 1.25) and is reached quicker in 35 ^∘^C in comparison to 25 ^∘^C.

**Fig. 7:**
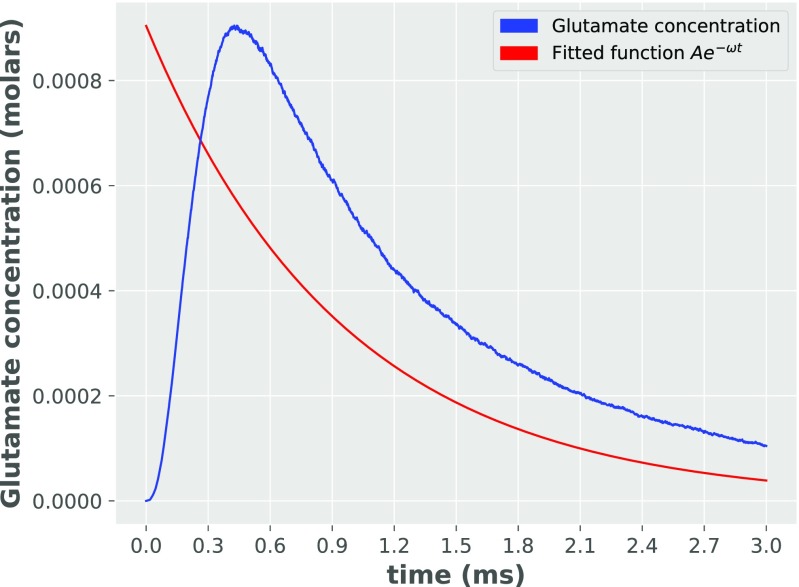
Glutamate concentration obtained from Monte Carlo simulation in 25 ^∘^C and assumed fitting curve proportional to *e*^−*ωt*^

However, the analytical model predicts a too rapid rise-time of conductance in comparison to experimental data (see the time of peak on Fig. 6 and on Fig. 1B of Postlethwaite et al. (2007)). This is due to the assumption (6) (see Methods). Namely, only a single exponential decay time (from a peak value at *t* = 0) of glutamate concentration was used - which only roughly approximates reality (see Fig. 7). However, we did not include any other, more complex glutamate concentration functions as they did not allow for closed-form analytical solution to the differential equations system including higher sub-conductance states.

**Fig. 8:**
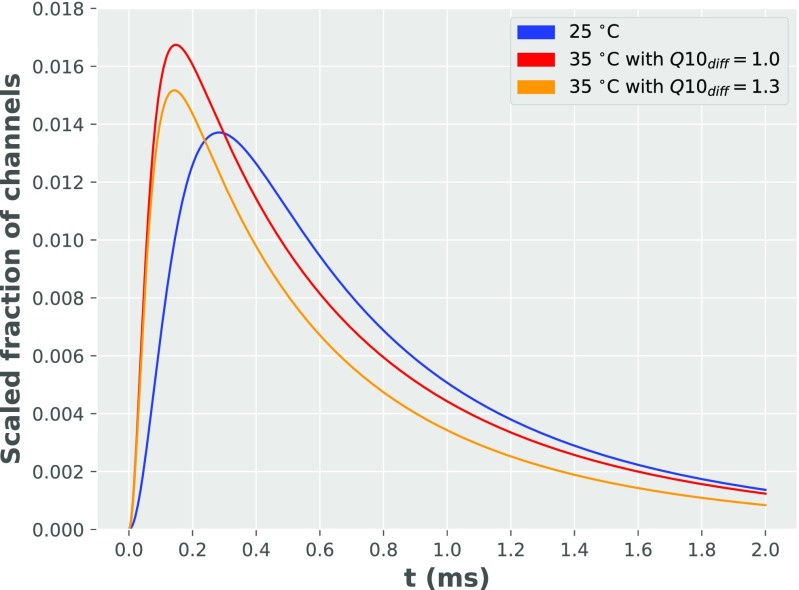
Influence of different *Q*_10_ diffusion coefficients on synaptic conductance

It was found that modifying the diffusion rates of the neurotransmitters by the temperature coefficient cannot increase the accuracy of the solution (in comparison to the experimental data) - as presented in Fig. 8. $Q_{10_{\text {diff}}}$ coefficient of diffusion (effectively multiplying diffusion coefficient of glutamate) with temperature, leads to quicker decay of glutamate concentration in the synaptic cleft and quicker rise time and decay of synaptic conductance. However, increasing $Q_{10_{diff}}$ causes also a smaller AMPAR peak conductance than is observed experimentally. This supports the previous conclusion of Postlethwaite et al. (2007) for the predominant role of postsynaptic kinetics in mediating temperature effects on synapses. In turn, this result may be important in the context of possible medical applications. Namely, the creation of a drug capable of altering receptor kinetics may lead to successful prevention of adverse temperature effects on synapse dynamics.^5^

**Fig. 9:**
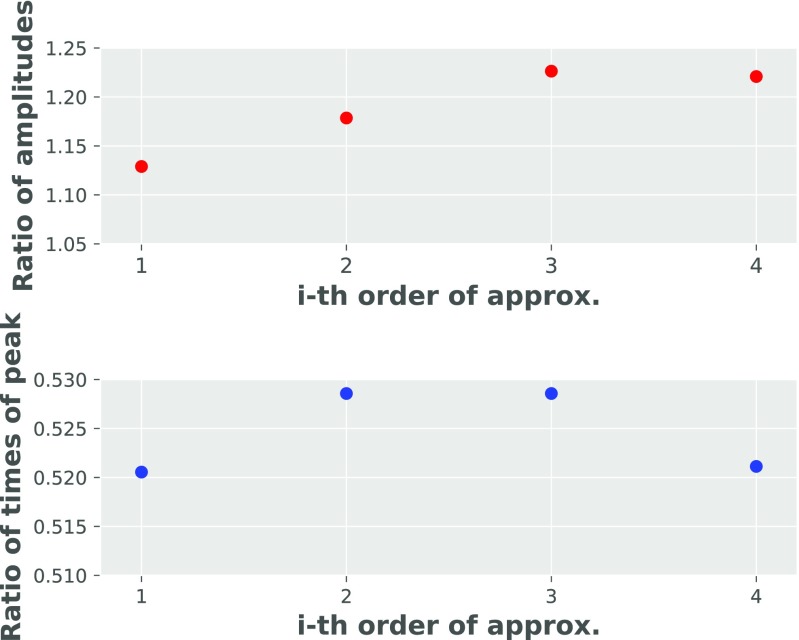
Comparing ratio of peak amplitudes and peak times 35 ^∘^C and 25 ^∘^C for different order approximations of higher sub-conductance states

The significance of including higher subconductance states in the analytical model was also investigated. It turned out, that including higher and higher states of subconductance leads to saturating increase of ratio (in 35 ^∘^C relatively to 25 ^∘^C) of synaptic conductance peak amplitudes (for about 12% in comparison to first order approximation), with minor influence on ratio of times of peak (Fig. 9). Furthermore, it was found that it is possible to achieve same dynamics of AMPAR synaptic conductance (qualitatively and quantitatively) by using 3rd approximation (without including 4-th order) and changing the fraction of the peak conductance at the 4-fold bound state for the 3rd state from 0.7 to 0.9. Therefore, we suppor the conclusion by Postlethwaite et al. (2007), which claims that higher temperature leads AMPARs to higher conducting states (thus increasing conductance peak amplitude).

The significance of including higher sub-conductance states in the analytical model was also investigated. Including higher states of sub-conductance leads to a saturating increase (in 35 ^∘^C relative to 25 ^∘^C) of synaptic conductance peak amplitudes (an increase of 12*%*, in comparison to the first order approximation), with only a minor influence on the ratio of times to peak (Fig. 9). Furthermore, it was found that it is possible to achieve the same dynamics of AMPAR synaptic conductance (qualitatively and quantitatively) by using the 3rd order approximation (but not the 1st or 2nd order) and changing the fraction of the peak conductance at the 3rd order open state from 0.7 to 0.9. Therefore, we support one of the results of Postlethwaite et al. (2007), which claims that higher temperature drives AMPARs to higher sub-conductance states (rather than only increasing unitary subconductances) cause the increase in conductance peak amplitude).

## Discussion

### **Normalization of fraction of channels**

Without any correction, the model would not be able to fit experimental data quantitatively (because the sum of all of the fractions of channels in different states does not equal 1). The reasons are the assumptions (3) and (4): the fraction of channels in state *C*_*i*_ for the (i + 1)-th order mesh equals 1. To resolve this problem, we introduce a normalization constant, which scales the analytical model output to agree with the results of detailed Monte Carlo simulation.

**Fig. 10:**
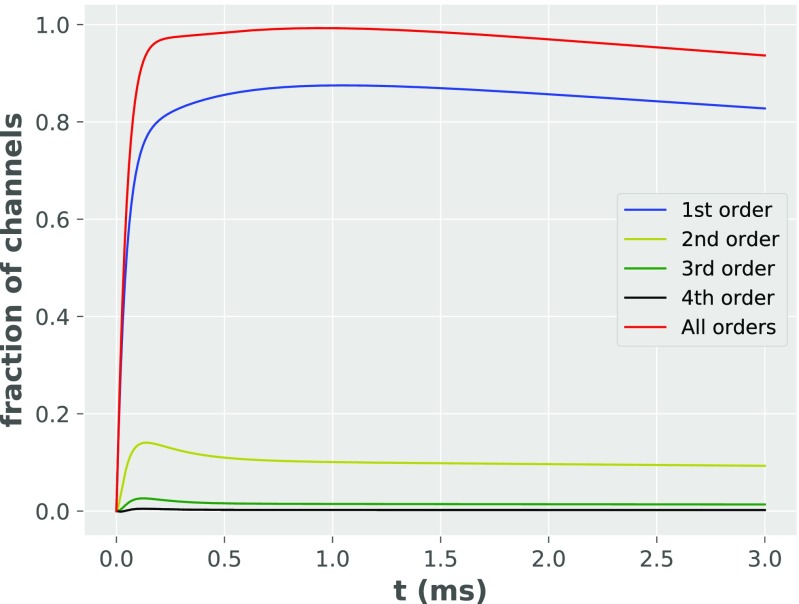
Fraction of bound AMPAR channels for different orders of kinetic Scheme 2

First, we calculated the sum of all bound channels in the analytical model. The results are presented in Fig. 10. The sum of all bound channels firstly rapidly increases and then after a time of approximately 0.5 ms gradually decreases over time. This is due to resensitization, which drives AMPARs to the C0 state (as assumed in Scheme 2). Additionally as the concentration of the glutamate in the cleft is low, the binding rate is substantially lower than unbinding rate, so no new channels get bound.

Second, we investigated what is the absolute number of the bound channels in the Monte Carlo simulation over time. The absolute number of unbound channels (easily convertible for the absolute number of bound channels as they sum up to one) is presented in Fig. 4. Finally, we compared the absolute number of bound channels (in Monte Carlo simulation) at the peak of the synaptic conductance (0.5*ms* after stimulation) with the sum of all bound channels (in the analytical model) at the respective peak (0.3*ms* after stimulation). A fraction of 0.13 of the channels is bound at the peak in the Monte Carlo simulation, which corresponds to ≈ 1 according to the analytical model. Therefore, to quantitatively reproduce Monte Carlo results to a good level of approximation, we only have to multiply all of the fractions of channels in the analytical model by 0.13. The normalization factor is weakly dependent on the order of subconductance assumed - as the 1st order contributes by far the most to the sum of all bound channels.

### **Glutamate binding model**

An analytical model showed that, with the assumptions we made, it is possible to reproduce experimental and averaged numerical results of the Monte Carlo simulation using independent glutamate binding. However, considering the complexity of biological systems and the role of variability in their behavior, we do not argue that independent binding is a biological reality, but rather a reliable approximation for simulating aspects of AMPAR dynamics and temperature response. In Postlethwaite et al. (2007), using Monte Carlo simulation, it was shown that variability of both rise and decay time constants of AMPAR conductance as a function of the mEPSC peak amplitude was not successfully reproduced by an independent binding model for the kinetic scheme they proposed. Although it is not possible to research this relationship using our differential-equation based model which describes only the averaged dynamics of the system, we suggest that the kinetic model proposed here (kinetic scheme in Fig. 2) may reproduce some aspects of the biological variability observed experimentally. To verify this we have run three sets of Monte Carlo simulations (with assumptions discussed in the Methods section) - two for kinetic scheme proposed by Postlethwaite et al. (2007) (one with cooperative and one with independent binding) and one set with the kinetic scheme proposed in this paper (with independent binding). The rate constants of the models were fitted to reproduce average behavior for the cooperative binding of Postlethwaite et al. (2007). We found that for the Scheme 2 (Fig. 2) with independent binding *skewness* = 0.62 and *CV* = 0.27 are close to the values resulting from kinetic scheme by Postlethwaite et al. (2007) with cooperative binding (*skewness* = 0.53, *CV* = 0.31) unlikely to kinetic scheme by Postlethwaite et al. (2007) with independent binding(*skewness* = 0.25, *CV* = 0.19).

### **Temperature dependence of AMPA receptor conductance**

Our simulations show that, for a single synapse, increased temperature causes larger peak amplitude of AMPAR conductance, which is also achieved quicker (the conductance peaks faster in time). However, these results are not easily interpretable in a more general context. One of the examples is predicting an influence of temperature-modified synaptic conductance on temporal summation of signals across morphology of a neuron. As a single synaptic conductance has a larger peak amplitude in higher temperature, a smaller number of EPSPs should elicit an action potential at the higher temperature. However, rise and decay time constants of the AMPAR conductance function are quicker at the higher temperature, so the effective summation window of the different signals should be shorter and therefore they should sum up less efficiently. These opposing features of AMPAR synaptic conductance hinder a simple description of compounded temperature effects on multi-synaptic networks: it is difficult to strictly predict (because we do not know the relative importance of amplitude and time constants of synaptic conductance curve) how temperature may influence temporal summation of the synaptic signal. In the future, more detailed study on this topic may allow us to investigate temperature effects from the level of single synapses to that of large neural networks, which may help in better understanding of complex and paradoxical field interactions in brain imposed by temperature (Andersen and Moser 1995). Additionally, detailed investigation of the influence of temperature on field potentials may be important in the context of temperature-sensitive epilepsy (Traub and Wong 1982).

### **Uncoupling assumption accuracy**

**Fig. 11:**
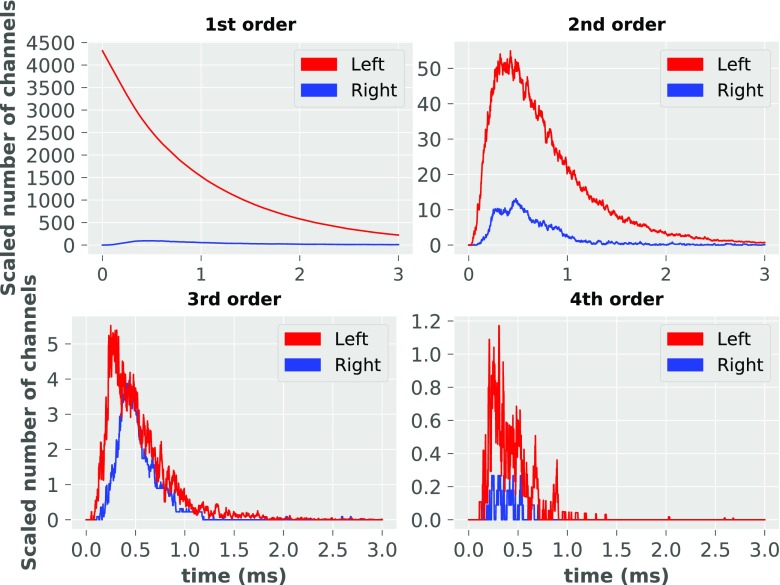
Uncoupling assumption validation for different orders of kinetic scheme. ‘Left’ and ‘Right’ denote for numerical values of left and right side of equation (1) respectively

To further test the accuracy of our uncoupling assumptions of differential equations in the analytical model, we carried out additional Monte Carlo simulation of synaptic transmission for the kinetic scheme proposed here, by comparing the dynamics of Scheme 2 without uncoupling, to the dynamics with uncoupling (Scheme 3). This comparison illustrated that the uncoupling assumption is fulfilled with different accuracy for each order (Fig. 11).

The assumption is best fulfilled for the first order of kinetic scheme (see Fig. 2) and the error associated with this order of sub-conductance is < 0.5*%*. For the second and fourth orders, the error is around 15*%*. The worst accuracy is for the third order, with an error level of roughly 40*%* at the peak of synaptic transmission. However, from (1), we may see that the accuracy of the uncoupling assumption is dependent also on a glutamate concentration: the higher the glutamate concentration is at PSDs, the better the accuracy of the assumption. Therefore these error percentages should be divided by a factor of about 1.8, as the glutamate concentration function is not able to correctly capture the dynamics of glutamate concentration in the synaptic cleft (see Fig. 7).

### **New modeling method of temperature effects on AMPA receptor**

The creation of an analytical model for the AMPA type synapse, capable of simulating temperature effects, has few potential applications. First, our analytical model was validated with experimental data and it may be implemented with high accuracy and efficiency in large neural network simulations, which (when combined with previous studies about temperature-dependence of the conductance of voltage-gated ion channels) may open new possibilities of researching temperature influences on neural dynamics in computational neuroscience. Second, due to the generality of this model, it is weakly dependent on the kinetic scheme or phenomenological method of synaptic conductance modeling we have chosen. The model provides simple theoretical linking between some models created in one temperature to any other (in a reasonable physiological range), by using the model developed here as a ‘linking bridge’, without performing additional experiments (however its accuracy has to be further carefully tested).

**Fig. 12:**
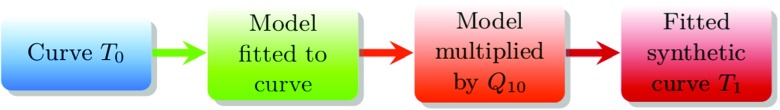
Scheme 4

This theoretical linking could be done as follows:

1. To some synaptic conductance curve, we fit (with free parameters being rate constants and glutamate concentration constants *A*, *ω* (with the constraints for their values being in physiological range) of the model developed here.
2. In fitted model, to determine dynamics at a different temperature, we multiply all of the AMPAR kinetic rate constants by an appropriate temperature dependent factor (*Q*_10_ = 2.4 for our simulations) and hence create a new synaptic conductance curve.
3. Finally, we fit desired phenomenological model to the synaptic conductance curve we achieved in the previous step (see also Scheme 4 on Fig. 12).

This approach shall be insensitive to possible fitting parameter degeneracy, due to the use of the common multiplication *Q*_10_ = 2.4 factor.

The model may be efficiently implemented in NEURON (Hines and Carnevale 1997) using NMODL (Hines and Carnevale 2000), making it ready for easy implementation in neural network simulations.

### **Comparison of the performance of the model with the simple AMPA kinetic models**

**Fig. 13:**
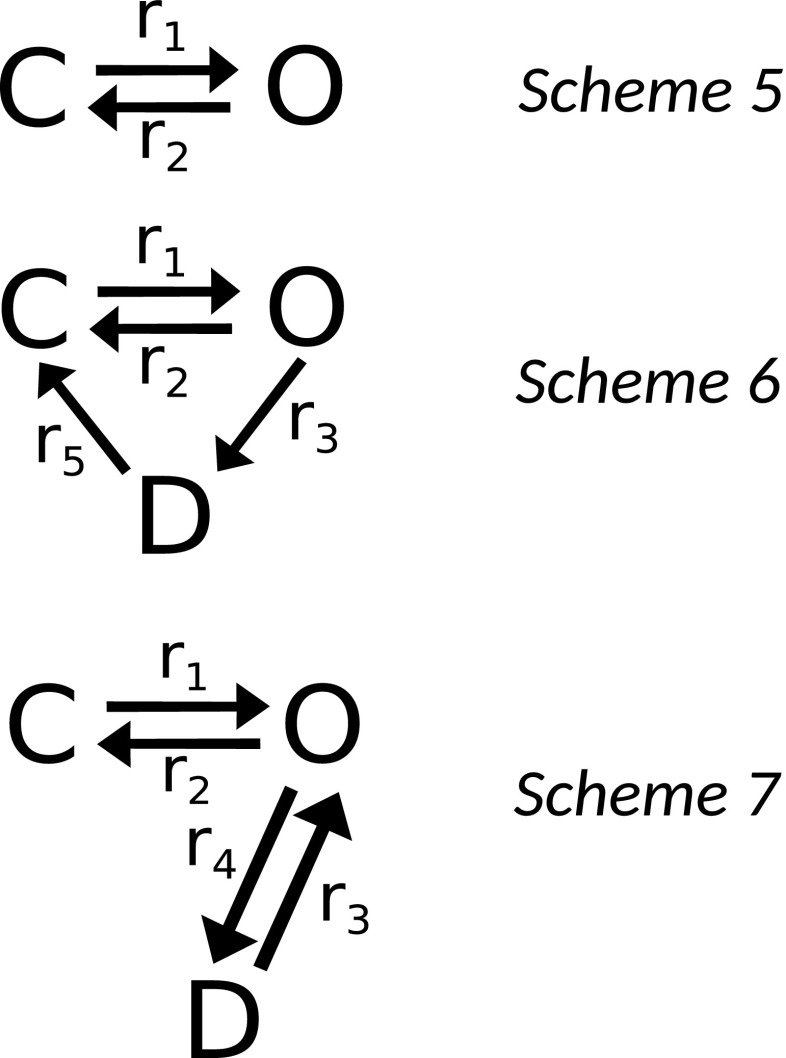
Kinetic schemes 5,6,7 (Destexhe et al. 1994b) used in the comparison of performance with the model developed in this paper

Set of the simple AMPA receptor kinetic models have been proposed previously by Destexhe et al. (1994b). Here, we will analyze three of the AMPA kinetic models proposed, which are shown in Schemes 5, 6 and 7 (Fig. 13). Employing the assumptions (1),(2),(5) and (6) (see Methods) we may solve the set of differential equations describing the kinetics of the AMPA receptor for a given kinetic model and include the influence of temperature by multiplication of the rate constants by *Q*_10_ coefficients (yet not assuming that all of the coefficients are equal to each other). However, temperature coefficients for the arbitrary kinetic scheme are not known *a priori* (as described earlier in the Introduction). Thus, we will find the temperature coefficients by fitting the rate constants of the simple models from Schemes 5, 6 and 7 to the analytical model (developed here) - this process is described in more detail in the previous paragraph. The full solutions of the coupled linear ODEs describing Schemes 5,6 and 7 are presented in Appendix B.

For kinetic models from Scheme 5 and 6 solutions may be discussed together as *r*_2_ and *r*_3_ rate constants are indistinguishable from each other (so we can reduce them to one constant). Models from Scheme 5 and 6 were unable to capture the dynamics of the AMPA receptor in different temperatures, as relatively large fitting error could hinder the subtle temperature dependence of the amplitude and time courses of synaptic conductance. For kinetic model from Scheme 7, it was possible to obtain accurate fit of the data in 25 ^∘^C. However, for the best fit in 35 ^∘^C the model was underfitting the amplitude of the synaptic conductance, which is crucial in the investigation of temperature dependence. The solution of the kinetic model from Scheme 7 needs additional constraints on parameters during the fitting process (as there are square roots in the solution). Moreover, the *Q*_10_ parameters used in this model do not have a physical meaning, as (for the best-fit) some of them were found to be lower than 1 (so they should decrease the speed of the conformational changes of AMPARs).

Therefore, without the more careful investigation of the simpler kinetic models, we propose that the analytical model developed here is a more convenient and accurate way to include temperature effects on AMPA receptor conductance.

## Conclusion

In the present study, an analytical model of an AMPA-type synapse including temperature effects was created. The model was developed on the bases of previous Markovian models describing the kinetics of the AMPA receptor and was simplified by uncoupling of the differential equation system, and by kinetic scheme modifications motivated by Monte Carlo simulation of synaptic transmission. This method may be used to make simple models of synaptic conductance easily-scalable for any temperature and may provide simple theoretical linking of neurobiological measurements (involving AMPA-type synapse) conducted in different temperatures. Due to its accuracy (in comparison to experimental data) and efficiency, this model may be used in large neural network simulations. This opens new possibilities to research various temperature effects on neural dynamics in large-scale multi-neuron experiments and simulations. It may provide a theoretical basis for better understanding of different neurological disorders associated with sub- and super- physiological temperatures. In conjunction with some previously proposed models (WaŻny and Wojcik 2014), this approach may shed some light on understanding paradoxical temperature influence in serious neurological disorders like autism spectrum disorder (Helt et al. 2008), which will be a scope of our interest in the forthcoming future.

## Appendix A

The solution of all order of differential equation system describing AMPA receptor kinetics:

$$\begin{array}{@{}rcl@{}} y_{scaled}(t)&=&0.1 A e^{-t (k_{c}+\omega)} k_{o} \left( -\frac{e^{t \omega} S}{R}+\frac{e^{(k_{c}-P) t}}{PR}+\frac{e^{k_{c} t}}{PS}\right) k_{b} + 0.4 A^{2} e^{-t (k_{c}+P + 2 \omega)} k_{o} \left( -\frac{e^{t (P+S+\omega)} (k_{c}-G)}{P R (P-\omega) (S-\omega)}-\frac{e^{t (k_{c}+\omega)} (\omega-S)}{R (P-\omega) (S-\omega) \omega}+\frac{e^{k_{c} t} (\omega-S)}{P (S-\omega) \omega (R+\omega)}\right.\\ &&+\left.\frac{e^{t (P + 2 \omega)} (\omega-P)}{R (P-\omega) (S-\omega) (R+\omega)}\right) {k_{b}^{2}}\\ && + 0.35A^{3} e^{-t (k_{c}+P + 3 \omega)}k_{o} \left( -\frac{2 e^{t (P+S+\omega)} (k_{c}-G) (-G+S-\omega) (S-G)}{P R (k_{c}-3 \omega) (P-2 \omega) (P-\omega) (R+\omega) (R + 2 \omega)}-\frac{e^{t (k_{c}+ 2 \omega)} (-G+S-\omega) (\omega-P) (S-G)}{R (P-2 \omega) (P-\omega) \omega^{2} (R+\omega) (R + 2 \omega)}+\frac{e^{k_{c} t} (k_{c}-G) (S-G)}{P R \omega^{2} (R+\omega) (R + 2 \omega)}\right.\\ &&-\left.\frac{2 e^{t (P + 3 \omega)}}{R (k_{c}-3 \omega) (R+\omega) (R + 2 \omega)}-\frac{2 e^{t (k_{c}+\omega)} (k_{c}-G) (-G+S-\omega)}{R (P-\omega) \omega^{2} (R+\omega) (R + 2 \omega)}\right) {k_{b}^{3}}\\ && +A^{4} e^{-t (k_{c}+P + 4 \omega)} k_{o} \left( -\frac{e^{t (k_{c}+ 3 \omega)} (S\,-\,G)(-G\,+\,S-\omega) (\omega\,-\,P) (3 \omega\,-\,G) (-G\,+\,k_{c}\,-\,3 \omega)}{6 R (P-3 \omega) (P-2 \omega) (P-\omega) \omega^{3} (R+\omega) (R + 2 \omega) (R + 3 \omega)}+\frac{e^{t (P+S+\omega)} (k_{c}\,-\,G) (S\,-\,G) (-G\,+\,S\,-\,\omega) (-G\,+\,k_{c}-3 \omega)}{P R (k_{c}-4 \omega) (P\,-\,3 \omega) (P\,-\,2 \omega) (P\,-\,\omega) (R\,+\,\omega) (R + 2 \omega) (R + 3 \omega)}\right.\\ &&+\left.\frac{e^{t (k_{c}+ 2 \omega)} (k_{c}-G) (-G+S-\omega) (-G+k_{c}-3 \omega)}{2 R (P-2 \omega) \omega^{3} (R+\omega) (R + 2 \omega) (R + 3 \omega)}\right.\\ && \left. -\frac{e^{t (k_{c}+\omega)} (k_{c}-G) (S-G) (-G+k_{c}-3 \omega)}{2 R (P-\omega) \omega^{3} (R+\omega) (R + 2 \omega) (R + 3 \omega)}-\frac{e^{t (P + 4 \omega)}}{R (k_{c}-4 \omega) (R+\omega) (R + 2 \omega) (R + 3 \omega)}+\frac{e^{k_{c} t} (k_{c}-G) (S-G) (-G+S-\omega)}{6 P R \omega^{3} (R+\omega) (R + 2 \omega) (R + 3 \omega)}\right) {k_{b}^{4}} \end{array} $$

where, *S* = *k*_*c*_ − *ω*, *R* = −*k*_*c*_ + *k*_*d*_ + *k*_*o*_ + *k*_*u*_, *P* = *k*_*d*_ + *k*_*o*_ + *k*_*u*_ − *ω*, *G* = *k*_*d*_ + *k*_*o*_ + *k*_*u*_

## Appendix B

Solution of the system of two coupled ODEs described by kinetic model of Destexhe et al. (1994b) (Scheme 5):

$$y(t)=\frac{B r_{1} e^{-r_{2} t} \left( e^{t (r_{2}-\omega)}-1\right)}{r_{2}-\omega} $$

where *B* is a constant.

Solution of the system of two coupled ODEs described by kinetic model of Destexhe et al. (1994b) (Scheme 6):

$$y(t)=\frac{B r_{1} e^{t (-(r_{2}+r_{3}))} \left( e^{t (r_{2}+r_{3}-\omega)}-1\right)}{r_{2}+r_{3}-\omega} $$

Solution of the system of two coupled ODEs described by kinetic model of Destexhe et al. (1994b) (Scheme 7):

$$\begin{array}{@{}rcl@{}} y(t)\!&=&\!\left( B r_{1} e^{-\frac{1}{2} t \left( \sqrt{(r_{2}+r_{3}+r_{4})^{2}-4 r_{2} r_{4}}+r_{2}+r_{3}+r_{4}\right)} \left( \small\left( \!-1 + e^{\sqrt{t((r_{2}+r_{3})^{2}+ 2(r_{3}-r_{2})r_{4}+{r_{4}}^{2})}} \small\right) r_{2} (r_{4}-\omega)\,-\,\left( r_{4} \left( -1 + e^{t \sqrt{(r_{2}+r_{3})^{2}+ 2(r_{3}-r_{2})r_{4}+{r_{4}^{2}}}} \right)\right.\right.\right.\\ &&+ \left( 1+e^{t \sqrt{(r_{2}+r_{3})^{2}+ 2(r_{3}-r_{2})r_{4}+{r_{4}}^{2}}} -2 e^{0.5 t(r_{2}+r_{3}+r_{4}+\sqrt{(r_{2}+r_{3})^{2}+ 2(r_{3}-r_{2})r_{4}+{r_{4}}^{2}})-2\omega)}\right) \sqrt{(r_{2}+r_{3})^{2}+ 2(r_{3}-r_{2})r_{4}+{r_{4}}^{2}}(r_{4}-\omega)\\ && \left.\left.- \left( -1+e^{t \sqrt{(r_{2}+r_{3})^{2}+(r_{3}-r_{2})r_{4}+{r_{4}}^{2}}} \right) r_{3}(r_{4}-\omega) \right) \right) \left/ \left( 2 \sqrt{-4 r_{2} r_{4}+(r_{2}+r_{3}+r_{4})^{2}}\right.\left( r_{2} r_{4} -(r_{2}+r_{3}+r_{4}) \omega + {\omega}^{2} \right) \right) \end{array} $$

## Footnotes

1
https://senselab.med.yale.edu/modeldb/showModel.cshtml?model=85981

2
https://senselab.med.yale.edu/modeldb/ShowModel.cshtml?model=239072

3 This approach is analogous to the way of including temperature effects on voltage-gated ion-channels (Hodgkin and Huxley 1952) and is motivated by the Arrhenius equation. Q10 is the multiplicative factor for increasing a given speed of reaction upon a 10 degree Celsius increase of temperature.

4 Departure from model based on neurotransmitter concentration occurring as a pulse (described by Dirac Delta at *t*_*p**u**l**s**e*_) proposed by Destexhe et al. (1994b) or Destexhe et al. (1994a) was motivated by availability of direct data of glutamate concentration on PSDs in Monte Carlo simulation and unit inconsistency problem (which is a result of modeling using Dirac Delta at *t*_*p**u**l**s**e*_ combined with the assumption about considering fraction of channels in *C*_0_ state as 1).

5 For example, a drug for slowing down the kinetics in a state of the brain hyperthermia - or a drug for speeding up kinetics in case of hypothermia.
